# Long non-coding RNAs in lung cancer: implications for lineage plasticity-mediated TKI resistance

**DOI:** 10.1007/s00018-020-03691-9

**Published:** 2020-11-10

**Authors:** Tongyan Liu, Chencheng Han, Panqi Fang, Hongyu Zhu, Siwei Wang, Zhifei Ma, Quanli Zhang, Wenjia Xia, Jie Wang, Lin Xu, Rong Yin

**Affiliations:** 1 Department of Thoracic Surgery, Jiangsu Cancer Hospital and Jiangsu Institute of Cancer Research and The Affiliated Cancer Hospital of Nanjing Medical University, Jiangsu Key Laboratory of Molecular and Translational Cancer Research, Collaborative Innovation Center for Cancer Personalized Medicine, Nanjing, China; 2 The Fourth Clinical College of Nanjing Medical University, Nanjing, China; 3 Department of Scientific Research, Jiangsu Cancer Hospital and Jiangsu Institute of Cancer Research and The Affiliated Cancer Hospital of Nanjing Medical University, Jiangsu Key Laboratory of Molecular and Translational Cancer Research, Nanjing, China; 4 Jiangsu Biobank of Clinical Resources, Nanjing, 210009 China; 5 Department of Clinical Pharmacy, School of Basic Medical Sciences and Clinical Pharmacy, China Pharmaceutical University, Nanjing, 210009 China

**Keywords:** Non-small-cell lung cancer, Tyrosine kinase inhibitors, Long non-coding RNAs, Lineage plasticity

## Abstract

The efficacy of targeted therapy in non-small-cell lung cancer (NSCLC) has been impeded by various mechanisms of resistance. Besides the mutations in targeted oncogenes, reversible lineage plasticity has recently considered to play a role in the development of tyrosine kinase inhibitors (TKI) resistance in NSCLC. Lineage plasticity enables cells to transfer from one committed developmental pathway to another, and has been a trigger of tumor adaptation to adverse microenvironment conditions including exposure to various therapies. More importantly, besides somatic mutation, lineage plasticity has also been proposed as another source of intratumoural heterogeneity. Lineage plasticity can drive NSCLC cells to a new cell identity which no longer depends on the drug-targeted pathway. Histological transformation and epithelial–mesenchymal transition are two well-known pathways of lineage plasticity-mediated TKI resistance in NSCLC. In the last decade, increased re-biopsy practice upon disease recurrence has increased the recognition of lineage plasticity induced resistance in NSCLC and has improved our understanding of the underlying biology. Long non-coding RNAs (lncRNAs), the dark matter of the genome, are capable of regulating variant malignant processes of NSCLC like the invisible hands. Recent evidence suggests that lncRNAs are involved in TKI resistance in NSCLC, particularly in lineage plasticity-mediated resistance. In this review, we summarize the mechanisms of lncRNAs in regulating lineage plasticity and TKI resistance in NSCLC. We also discuss how understanding these themes can alter therapeutic strategies, including combination therapy approaches to overcome TKI resistance.

## Introduction

Lung cancer is the leading cause of cancer-associated mortality worldwide, with non-small-cell lung cancer (NSCLC) as the main histological subtype with a poor 5 year survival [1]. Improved understanding of the molecular classification of lung cancer has revolutionized the treatment of NSCLC. Almost two-thirds of patients with NSCLC are oncogene addicted, approximately half of whom are exquisitely sensitive to targeted therapies [2, 3]. These include activating mutations or fusions in epidermal growth factor receptor (EGFR), serine/threonine-protein kinase b-raf (BRAF), anaplastic lymphoma kinase (ALK), and ROS1 receptor tyrosine kinase [3].

Although agents that target the tyrosine kinase domain of these oncogenes improve clinical outcomes of patients with NSCLC, responses to these drugs are generally temporary and limited by emergence of resistance [4]. Resistance to tyrosine kinase inhibitors (TKI) are generally associated with acquired somatic mutations, including genetic alterations that enable bypassing target inhibition through ineffective binding of the drugs, as well as activation of collateral or alternative survival pathways [3, 5, 6]. In addition to these genetic mechanisms of drug resistance, non-mutational mechanisms termed lineage plasticity are also associated with TKI resistance. Lineage plasticity refers to as the ability of cells transferring from one committed developmental pathway to another. It enables the adaptation and survival of tumors under adverse conditions including hypoxia and targeted therapies. Therefore, it is proposed as a mechanism of tumor cells escape from targeted dependency. Lineage plasticity can be both dependent on and a driver of intratumoral heterogeneity [7, 8]. In contrast to genetic mechanisms of TKI resistance, lineage plasticity-associated resistance primarily relies on phenotype switching, with one single genotype gives rise to different phenotypes upon drug treatment. It enables cancer cells to reversibly convert to new or hybrid lineages that is independent of TKI-targeted pathway [9].

Data from single-cell profiling and lineage tracing technologies suggest that a single cell can give rise to multiple states. Mechanistically, epigenetic modifications and transcriptomic transitions may mediate the emergence of new cell states. Long non-coding RNAs (lncRNAs) the dark matter of the genome, are capable of regulating variant malignant processes of NSCLC through epigenetic modification [10]. Importantly, lncRNAs often offer distinct advantages over proteins for some forms of epigenetic regulation [11]. Recent studies show the transcriptional heterogeneity regulated by lncRNAs might drive the phenotypic switch from one histological category to another, contributing to the lineage plasticity-mediated TKI resistance.

Targeting lineage plasticity provides a new opportunity to prevent the emergence of drug-tolerant cell states and enables TKI to achieve deeper responses. In this review, we performed a literature review covering the publication of the last 15 years on the topic of “long non-coding RNAs regulated lineage plasticity in lung cancer”. The keywords used for searching were “cell plasticity”, “cancer” and “non-coding RNAs”. We summarize current understanding of lineage plasticity as a mechanism of TKI resistance in NSCLC and discuss the implications of lncRNAs in lineage plasticity. Finally, we also discuss potential therapeutic strategies to circumvent lineage plasticity in NSCLC.

## Lineage plasticity in response to targeted therapies

**Fig. 1:**
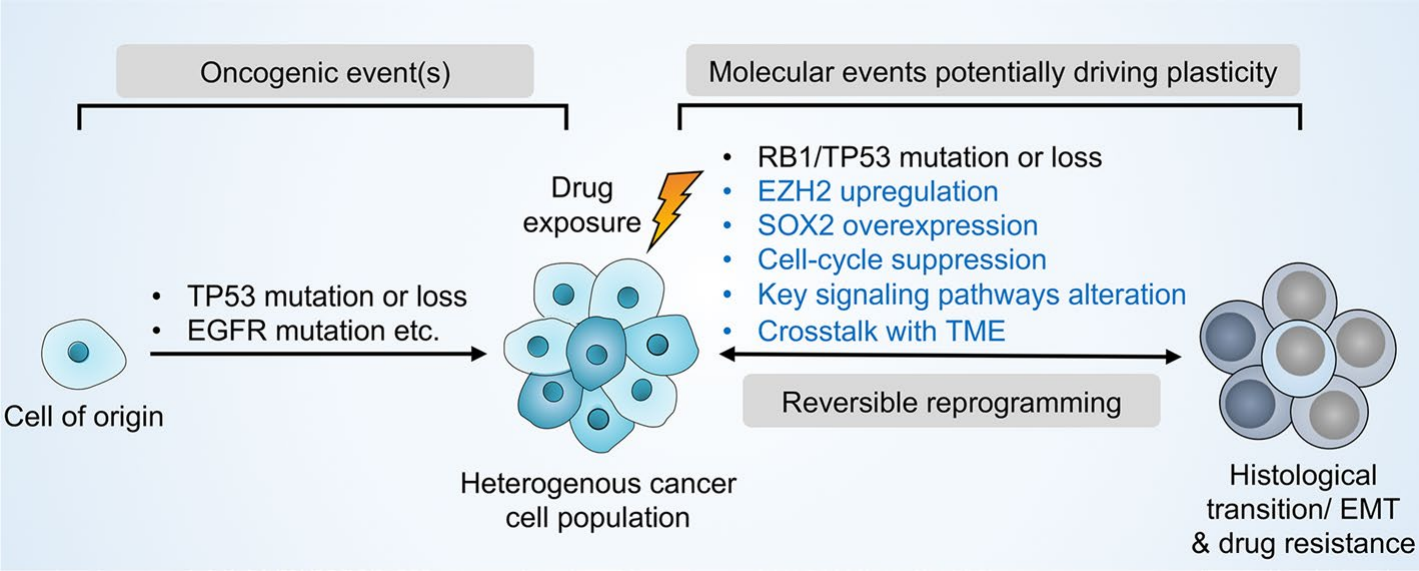
Lineage plasticity lead to TKI resistance in NSCLC. Upon targeted therapies, various molecular events can promote lineage plasticity, thereby driving intratumoural heterogeneity and drug resistance. RB1 and TP53 mutation or loss NSCLC cells are more likely to transdifferentiate into small cell tumors. Increased histone-modifying enzymes, such as enhancer of zeste homologue 2 (EZH2) and lineage-associated transcription factors, such as SOX family genes mediates the reprogramming of NSCLC into slow-cycling, drug-tolerant cell states. These slow-cycling, drug-tolerant cells generally present neuroendocrine differentiation and epithelial-to-mesenchymal transition (EMT). Alterations of key signaling pathways and crosstalk with the tumor microenvironment also control lineage plasticity. Collectively, the plasticity-permissive molecular environment under the pressure of targeted therapies trigger the intratumoural clones presenting an alternative histology to that initially diagnosed, which might become the predominant cell type and exhibit drug resistance. Blue font: lncRNA-mediated molecular events that promote lineage plasticity

Lineage plasticity, the ability of cells to reversibly transfer from a certain developmental pathway to another, has been regarded as a source of intratumoral heterogeneity and of tumor adaptation to adverse tumor microenvironment such as hypoxia and exposure to targeted therapies [7]. Histological transformation of adenocarcinomas to neuroendocrine and epithelial–mesenchymal transition are two well-known phenotypes of lineage plasticity in NSCLC upon TKI treatment [7, 12]. NSCLC cells has been reported to hijack developmental process involved in epigenetic modification and transcriptional reprogramming to become phenotypically plastic, and reversibly transform into one or several drug-tolerant cell identities [13, 14]. Interestingly, the emergence of slow-cycling tumors cells is primarily considered as the first state before histological transformation and further reprogramming into a drug-resistant state [15, 16]. Adenocarcinomas with concomitant inactivation of RB1 and TP53 are more likely to transdifferentiate into neuroendocrine tumors in NSCLC [17–20]. Furthermore, loss of lung lineage-specifying transcription factor gene Nkx2-1, overexpression of lineage-specific transcription factors such as SOX genes, and dysregulation of epigenetic regulators such as histone methyltransferase, changes of key signaling pathways (IL-6-STAT3 or RXR, etc.), as well as interactions with tumor microenvironment (TME) have been associated with intratumoural heterogeneity and lineage plasticity-mediated drug resistance [7, 14] (Fig. 1).

### Histological transformation

Histological transformation also referred to as transdifferentiation, which presents the process that cells convert from one lineage to another [21]. Targeted therapy-induced neuroendocrine transdifferentiation has been particularly described in NSCLC and prostate cancer [12, 22]. The transformation of EGFR-mutant adenocarcinoma to a SCLC histology was first reported in a 45-year-old woman with EGFR-mutant adenocarcinoma who underwent erlotinib treatment for 18 months before relapse with the features of SCLC, and positive immunostaining of neural cell adhesion molecule 1 (NCAM1), chromogranin A and synaptophysin [23]. In rare cases, NSCLC could adopt to other neuroendocrine histology, for instance, large cell neuroendocrine carcinoma and small and large cell carcinoma [19]. Transformation to sarcomatoid carcinoma has also been observed in ALK-rearranged NSCLC in resistant to crizotinib. Importantly, the transformed SCLC tumor samples maintained their original mutation [23], which indicates these tumors were emerging from the original adenocarcinoma, rather than de novo cancers. Genetically, NSCLC cells with RB1 and TP53 loss are more likely to transdifferentiate towards a neuroendocrine identity upon TKI treatment [17, 18]. Similar observations were made in castration-resistant prostate cancer (CRPC), with approximately one-quarter of CRPC acquire androgen receptor (AR) independent resistant through phenotypic switching [22]. These AR-independent CRPC tumors are referred to as neuroendocrine prostate cancer (NEPC) [22].

### Epithelial–mesenchymal transition

Epithelial–mesenchymal transition (EMT) is another type of tumor cell plasticity related to TKI resistance [12, 24], which tumor cells lose their epithelial features and acquire cellular alterations favoring more invasive, mesenchymal properties. Mesenchymal characteristics were observed in vitro and in vivo EGFR-mutant lung cancer models that acquired resistance to first-generation EGFR-TKI with no genetic alterations [25, 26]. Moreover, tumor biopsy samples from EGFR-TKI resistance patients presented increased vimentin expression and downregulated E-cadherin expression compared with tumor tissues taken before TKI treatment. Importantly, tumor cells undergo epithelial–mesenchymal plasticity retain their original mutation spectrum, indicating EMT as a mechanism of TKI resistance [12, 27]. The precise mechanism of epithelial–mesenchymal plasticity remains to be elucidated. Increased levels of pleiotropic signaling factors, such as transforming growth factor-β (TGF-β), epidermal growth factor (EGF), hepatocyte growth factor (HGF), NOTCH, fibroblast growth factor (FGF) and WNT ligands can initiate a signaling cascade resulting the expression of EMT transcription factors [13]. Furthermore, reduced expression of proapoptotic proteins such as PUMA [28] and increased drug efflux [29] have also been reported to associate with EMT process upon TKI treatment.

## Deregulated lncRNAs involved in lineage plasticity upon targeted therapies

### Neuroendocrine transdifferentiation-associated lncRNAs

Clonal analysis showed that complete loss of both RB1 and TP53 may predict neuroendocrine transdifferentiation in NSCLC upon EGFR-TKI treatment [18]. However, cell lines and mouse model studies of lung and prostate cancer suggested that inactivation of RB1 or TP53 alone is not sufficient for their histological transformation or effect their sensitivity to EGFR-TKI [30–32]. Additionally, epigenetic states, specially, lncRNAs are involved in cell-fate determination [33]. Crea et al. identified the first NEPC-associated lncRNA-MIAT, which contributed to the neuroendocrine transdifferentiation of CRPC [34]. Moreover, Ramnarine et al. identified lncRNAs FENDRR, H19, LINC00514, LINC00617, and SSTR5-AS1 to be implicated in the development of NEPC [35]. Altogether, these data suggest lncRNAs can be strong candidate for clinical biomarkers and therapeutic targets in preventing neuroendocrine transdifferentiation in CRPC. Nevertheless, lncRNAs associated with neuroendocrine transformation in NSCLC in response to TKI treatment warrant further investigation. (Table 1).

**Table 1:** Examples of lncRNAs implicated in lineage plasticity in NSCLC

lnRNA	Association with Cancer	Function	Cellular phenotypes	Refs
Slow cycling				
lincRNA-p21, LED	p53-induced lncRNAs	Protein interactions (interaction with hnRNPK to regulate CDKN1A; interaction with CKDN1A enhancer	Cell-cycle arrest	[61]
LINC-PINT, TUG1, PR-lncRNA-1, PR-lncRNA-10	p53-induced lncRNAs	Connects p53 pathway with epigenetic silencing by PRC2	Cell-cycle arrest	[61]
p15-AS1, MIR31HG, MIR100HG	Downregulated in lung cancer	Silencing pf p15 through heterochromatin formation; Protein interactions (decoy for polycomb group proteins to repress INK4A transcription)	Growth arrest and apoptosis	[65, 66]
MALAT1, CASC2, TINCR	Downregulated in lung cancer	Chromatin condensation by recruitment of PRC2	Growth arrest	[68]
EMT				
LINC00673, CAR10, XIST, LINC81507, LINC TTN-AS1, LINC00858, H19, SOX20T, LINC00483, PRNCR1, SNHG6, ATB, PNUTS	Overexpressed in lung cancer	miRNA decoy (for miR200, miR181a)	EMT, invasion, proliferation and metastasis, drug resistance	[35]
ELIT-1, TBILA, LINP, HOTAIR,NKILA and LINC001186	TGFβ-regulated lncRNAs	Interacts with various proteins; interferes with different signaling pathway	EMT, metastasis	[45–48, 56, 57]
MEG3	Overexpressed in NSCLC cells	Chromatin condensation by recruitment of EZH2 to silence CDH1 and miR-200 family	EMT, metastasis	[50]
MALAT1	Overexpressed in lung cancer	Chromatin condensation by recruitment of EZH2 to silence E-cadherin	EMT, metastasis	[51]
FEZF1-AS1	Overexpressed in lung cancer	Chromatin condensation by recruitment of LSD1 and EZH2 to repress E-cadherin	EMT, metastasis	[52]
HOXA11-AS1	Overexpressed in lung cancer	Gene methylation by recruitment of EZH2 and DNMT1 to inactivate miR200b	EMT, metastasis	[53]
NORAD	Overexpressed in lung cancer	Molecular scaffold for proteins (importin β1 and Smads)	EMT, metastasis	[49]
Epigenetic reprogramming				
HOXA11-AS	Overexpressed in NSCLC	Chromatin condensation by recruitment of PRC2 to silence miR200	EMT, tumor progression	[53]
FBXL19-AS1	Overexpressed in LUAD	miRNA Decoy (for miR-203a-3p), enhances E2F and ZEB1	Proliferation, migration and metastasis	[76].
Reprogramming transcription factors				
SOX2-OT	Overexpressed in lung cancer	Regulation the expression of SOX2	Neural differentiation	[81, 82]
Key signaling pathways				
SNHG1	Overexpressed in cisplatin-resistant NSCLC	miRNA Decoy (for miR140-5p), increases WNT/β-catenin pathway	Neural differentiation, drug resistance	[90]
LINC00673	Overexpressed in LUAD	Molecular scaffold for proteins (DDX3 and CK1ε), promotes WNT/β-catenin signaling pathway	Cell migration, invasion and metastasis	[91]
NEAT1, FOXO2-AS1	Overexpressed in NSCLC	Upregulation of WNT/β-catenin signaling pathway	Acquisition of cancer stem cell-like properties, proliferation	[89, 92, 93]
MALAT1,H19	Overexpressed in NSCLC	miRNA decoy (for miR-124, miR-17, miR-29b-3p) to activate STAT3	EMT, proliferation, migration and invasion	[95–97]
TNK2-AS1	Overexpressed in NSCLC	Interaction with STAT3 to enhances its protein stability	Promotes angiogenesis	[98]
Tumor microenvironment				
GNAS-AS1	Overexpressed in NSCLC	Promote macrophage M2 polarization	Cell migration and invasion	[112]
XIST	Overexpressed in NSCLC	Promote macrophage M2 polarization	Increased the progression of lung cancer and TAM-mediated drug resistance	[113]

*NSCLC* non-small cell lung cancer, *LUAD* lung adenocarcinoma, *TGFβ* transforming growth factor-β, *EZH2* zeste homologue 2, *LSD1* lysine-specific histone demethylase 1, *DNMT1* NDA methyltransferase 1, *YAP1* yea-associated protein 1, *SOX2* SRY-box transcription factor 2, *EMT* epithelial–mesenchymal transition, *TAM* tumor-associated macrophage

### EMT-associated lncRNAs

EMT is a dynamic process which tumor cells obtain phenotypic changes through epigenetic modifications [36]. Emerging evidence highlights the involvement of lncRNAs in EMT process in NSCLC [37].

Recently, a group of lncRNAs have been shown to promote drug resistance by acting as molecular decoys to sequester miRNAs associated with EMT. For example, LINC00673, CAR10, XIST, LINC81507, TTN-AS1, LINC00858, H19, SOX20T, LINC00483, PRNCR1, SNHG6, ATB and alternative splicing-generated lncRNA-PNUTS [38] are shown to sequester miRNAs from binding EMT associated genes, such as zinc finger E-box binding homeobox 1 and 2 (ZEB1 and ZEB2), thereby initiate EMT in NSCLC [39–46]. Increased levels of transforming growth factor-β (TGFβ) are reported to facilitate EMT via interleukin-6 (IL-6) in EGFR TKI-resistant NSCLC cells [47]. Several profiling studies have identified the roles of TGFβ-regulated lncRNAs such as ELIT-1, TBILA, LINP and HOTAIR in promoting EMT in NSCLC cell lines [48–51]. Mechanistically, TGFβ-activated lncRNA ELIT-1 can bound to Smad3 and improved Smad-responsive promoter activities by recruiting Smad3 to the promoters of its target genes, which include Snail, and ELIT itself. Thus, ELIT-1 forms a positive feedback loop to promote TGFβ/Smad3 signaling pathway and, therefore, promote EMT progression [48]. NORAD, a cytoplasmic long non-coding RNA is also reported to promote TGFβ-induced EMT in NSCLC cell lines [52]. It is shown to enhance the physical interaction of importin β1 with Smads, thereby promoting the nuclear accumulation of Smad complexes induced by TGFβ. Furthermore, many lncRNAs associate with epigenetic regulators to regulate EMT. For instance, lncRNA MEG3 is able to recruit EZH2 and epigenetically silencing CDH1 and miR-200 family in NSCLC cell lines [53]. Similarly, MALAT1 can also recruit EZH2 to silence E-cadherin and, therefore, induce EMT in NSCLC [54]. LncRNA-FEZF1-AS1 was shown to epigenetically repress E-cadherin by binding with lysine-specific histone demethylase 1 (LSD1) and EZH2 in NSCLC cells [55]. HOXA11-AS1 was reported to recruit EZH2 and DNA methyltransferase 1 (DNMT1) to the promoter of miR200b and inactivate miR200b, thereby promoting EMT in NSCLC [56]. In contrast, a number of epigenetic upregulated lncRNAs can inhibit EMT through histone modification in NSCLC [57, 58]. For example, lncRNA-BANCR inhibits EMT through histone acetylation [58]. Most TGFβ-regulated lncRNA were reported to promote EMT, however, TGFβ-regulated lnRNAs NKILA and LINC001186 are reported to inhibit EMT by suppressing the expression of Snail [59, 60]. Targeting EMT-associated lncRNAs may restore the sensitivity to TKI, further studies are needed. (Table 1).

## Mechanisms of lncRNA-mediated lineage plasticity

**Fig. 2:**
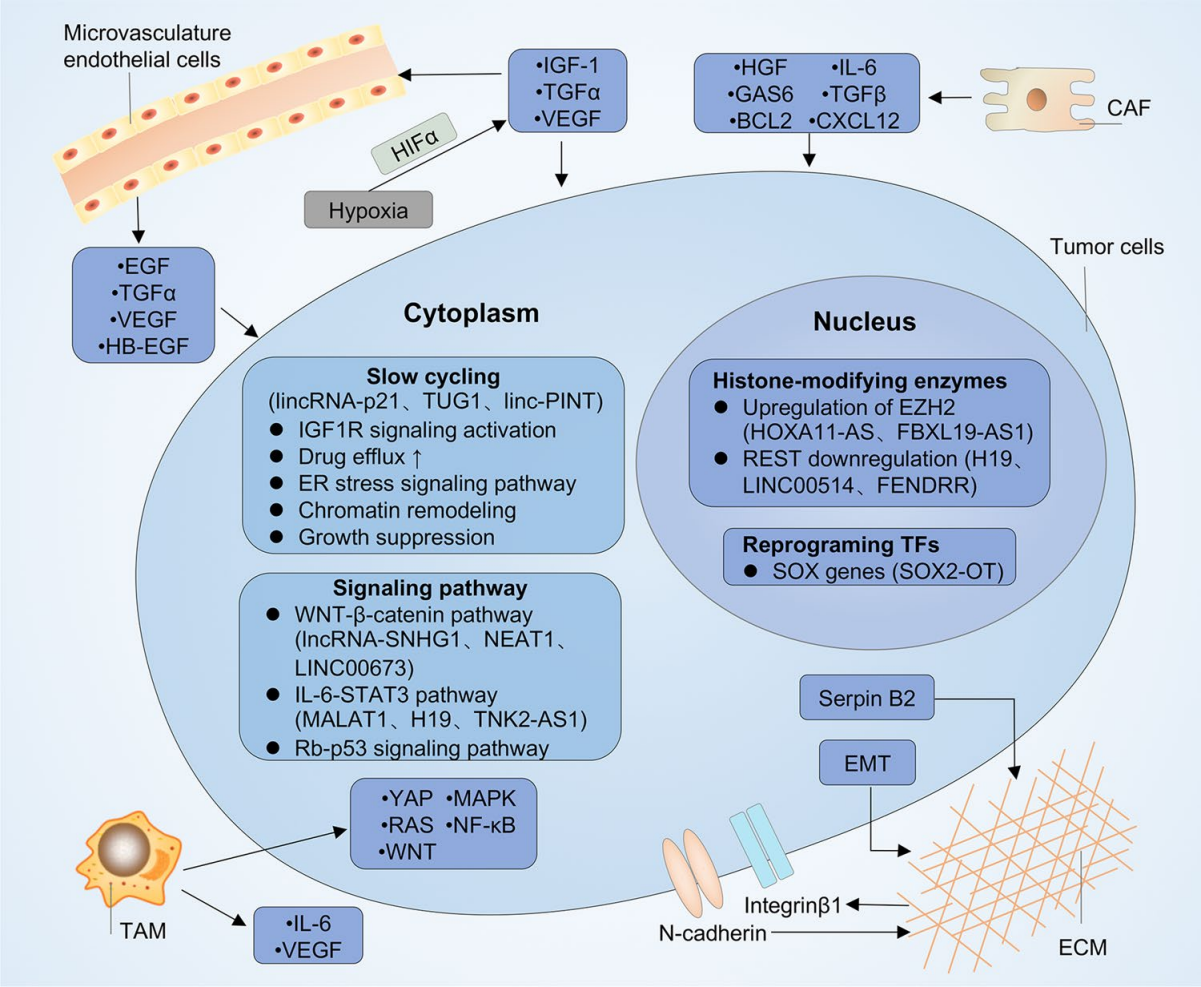
Mechanisms of lncRNA-mediated lineage transition. Transcription factors (such as SOX family), histone-modifying enzymes (such as enhancer of zeste homologue 2 (EZH2) and RE1-slienicng transcription factor (REST)) regulate the reprogramming tumor cells into slow-cycling, drug-tolerant states. LncRNAs such as lincRNA-p21, TUG1, linc-PINT, HOXA11-AS, SOX2-OT, FBXL19-AS1, LINC00514, FENDRR are implicated in the reprogramming process. Alterations in several key signaling pathways such as WNT-β-catenin pathway, IL-6/STAT3 pathway, NF-κB pathway and YAP pathway promote phenotypic switching upon TKI treatment. lncRNAs such as MALAT1, GHET1, SNHG1, NEAT1, H19 TNK2-AS1 are involved in these key signaling pathways. Crosstalk with the tumor microenvironment, through secretion of various cytokines from cancer-associated fibroblasts such as hepatocyte growth factor(HGF), growth arrest-specific protein 6 (GAS6), CXC-chemokine ligand 12 (CXCL12), interleukin-6 (IL-6) and transforming growth factorβ (TGFβ); cytokines from endothelial cells such as epidermal growth factor (EGF), transforming growth factor-α (TGFα), vascular endothelial growth factor (VEGF) and heparin-binding EGF-like growth factor (HB-EGF); Interluekin-6 (IL-6), VEGF from tumor-associated macrophage (TAM) controls tumor plasticity. The enhanced cell–cell adhesion via increased expression of integrin β1 and N-cadherin in tumor cells and increased extracellular matrix (ECM) stiffness via Serpin B2 can also promote tyrosine kinase inhibitors (TKI) resistance. LncRNAs involved in the M2 polarization of macrophage such as XIST and GNAS-AS1 played a part in TKI resistance. Lastly, low level of oxygen can active hypoxia-inducible factor 1α (HIF1α) in tumor cells, causing autocrine signaling by secretion of TGFα, VEGF and insulin-like growth factor (IGF-1), which promotes resistance to TKI therapy. *TFs* transcription factors, *CAF* cancer-associated fibroblasts

The molecular mechanisms underlying lineage plasticity-mediated TKI resistance remain to be fully established. However, tumor cells go through a slow-cycling drug-tolerant state is generally considered as the first step for lineage plasticity-mediated resistance. Moreover, epigenetic and transcription factor changes and alterations of key signaling pathways, as well as crosstalk with tumor microenvironment (TME) can favor the development of phenotypic switching and TKI resistance (Fig. 2).

### Emergence of slow-cycling cells

A drug-tolerant, slow-cycling state was first identified in bacteria [61]. In that scenario, a group of drug-tolerant slow-cycling bacteria survive in response to antibiotic treatment, and can further convert to a proliferative state and re-established drug sensitive phenotype upon drug withdrawal [61]. Similarly, reversible drug-tolerant slow-cycling persisters were reported in NSCLC. Notably, a stepwise transition may occur in NSCLC upon TKI treatment, tumor cells first reversibly enter a slow-cycling state, then regain proliferation and ultimately become drug-resistant through further epigenetic changes or via genetic modifications (such as EGFR-T790M) [6, 15]. More recently, Sanchez-Danes et al. also discovered that in basal cell carcinoma, drug-tolerant slow-cycling residuals cells can lead to relapse upon drug withdrawal [62]. Forcing these slow-cycling cells to proliferate enhanced their sensitivity to vismodegib treatment, which leaded to their elimination [62].

LncRNAs involved in regulating cell-cycling conditions in cancer are primarily defined as p53-related lncRNAs [63]. Genome-wide profiling of p53-regulated enhancer RNAs identified p53-induced lincRNA-p21, LINC-PINT, TUG1, PR-lncRNA-1, PR-lncRNA-10 and LED to support cell-cycle arrest [63]. Mechanistically, lincRNA-p21 bound to heterogeneous nuclear ribonucleoprotein K (hnRNPK) to regulate cyclin-dependent kinase inhibitor 1A (CDKN1A) is cis and arrest the cell cycle [64]. Another p53-induced lncRNA DINO was shown to interact with p53 protein and enhanced its stabilization, creating a p53 auto-amplification loop, thereby promoting damage signaling pathway and cell cycle arrest in the absence of DNA damage [65]. Moreover, alterations in cell cycle proteins, including the silencing of cyclin dependent kinase (CDK) inhibitor p16 and p15 are associated with resistance to EGFR TKI in NSCLC patients [66]. Several lncRNAs such as p15-AS-1 and MIR31HG can suppress p15 or p16 through heterochromatin formation [67, 68]. Moreover, MIR100HG encoded lncRNA interacts with HuR/ELAVL1 as well as several HuR-target genes (oncogenes) to suppress cell proliferation [69]. In addition, lncRNA TINCR, CASC2, MALAT1 were involved in FBXW7-mediated cell cycle arrest in various cancers [70]. Nevertheless, it remains to be elucidated whether these cell cycle-associated lncRNAs are implicated in the drug-tolerant refractory cells under the pressure of TKI. It is important to note that most of these studies are conducted in vitro, and should, in the future, be further validated in vivo. 

### Epigenetic modification

The histone methyltransferase EZH2, which is the catalytic subunit of Polycomb repressive complex 2 (PRC2), is reported to promote neuroendocrine transdifferentiation in a mouse model of lung cancer [71]. Similarly, EZH2 is also a well-established feature of NEPC [31, 72]. Pharmacological or genetic inhibition of EZH2 activity in NEPC cell lines can reverse its lineage conversion and regain drug sensitivity [30, 31, 73] RE1-slienicng transcription factor (REST), another epigenetic regulator, is found in lung and prostate cancer during neuroendocrine conversion [74, 75]. REST suppresses gene expression generally through recruiting the co-repressors such as EZH2 [75]. Elevated levels of REST are reported to promote neuroendocrine differentiation in EGFR TKI resistance NSCLC cell lines via Notch signaling pathway [76].

Many lncRNAs have been shown to scaffold and recruit epigenetic regulator to specific genome loci. For example, lncRNAs HOXA11-AS can recruit PRC2 complex to silence miR200b [56]. On the other hand, a few lncRNAs are regulated by E2F transcription factor 1 (E2F1), which is a transcription factor that induce EZH2 expression [77]. Transcription factor-binding site (TFBS) analysis identified lncRNAs FENDRR, H19, LINC00514, LINC00617 and SSTR5-AS1 include TFBS motifs for REST and E2F, and the expression of these lncRNAs were implicated in the development of NEPC [35]. Furthermore, FBXL19-AS1/miR-203a-3p axis was found to enhance E2F1 and ZEB1 in lung adenocarcinomas (LUAD) cells [78]. Therefore, lncRNAs are important players in scaffolding EZH2-dependent gene silencing and subsequent regulate EZH2-mediated phenotypic switching.

### SOX family members

Several lineage-specific transcription factors are involved in lineage plasticity and drug escape both in NSCLC and prostate cancer. The SOX family is important in regulating cell fate decisions and is implicated in phenotypic conversion in various cancer models [79]. For instance, the expression of SOX2 was increased in TP53 and RB1-deficient GEMMs and xenograft models of LUAD and prostate cancers [30, 31]. Moreover, the neural lineage-specific factor BRN2, which is specifically expressed in SCLC and NEPC tumors, mediates SOX2 expression and is key for neuroendocrine transformation [80, 81]. Furthermore, insulinoma-associated-1 (INSM1), which encodes a zinc-finger transcription factor, has recently emerged as a specific neuroendocrine transcription factor and a sensitive biomarker for neuroendocrine tumors [82].

LncRNA SOX2 overlapping transcript (SOX2-OT) are functionally assumed to be associated with neuronal like differentiation and carcinogenesis [83, 84]. Concordant expression of SOX2 and SOX2-OT is found in lung and breast cancer [83]. Notably, SOX2-OT can generate six transcript variants in different cancer models [85, 86], and have been proposed to play a role in regulating the expression of SOX2 [84, 85]. Collectively, SOX reprogramming factors, together with other lineage-associated transcription factors, are key for cellular plasticity in TKI resistant NSCLC cells. LncRNA-SOX2-OT, as SOX2 overlapping transcript also makes contribution for promoting the transition toward neural crest state in NSCLC. 

### Key signaling pathways

Several signaling pathways play a part in phenotypic switching upon TKI treatment. The activation of WNT-β-catenin pathway is reported to promote neuroendocrine differentiation in various cancer models [87]. Meder et al. discovered NOTCH-Achaete-scure complex homolog 1(ASCL)-WNT signaling pathway could inactivate RB by phosphorylation and, therefore, promote neuroendocrine differentiation in NSCLC [88, 89]. Moreover, achaete-scute homolog 1 (ASH1) also acts as a positive regulator of WNT/β-catenin pathway, transforming NSCLC into a SCLC phenotype with neuroendocrine features both in vitro and in vivo models [90]. A number of lncRNAs have been found to active WNT/β-catenin to induce EMT and therapy resistance in NSCLC [91–93]. For instance, lncRNA-SNHG1 can sequester miR140-5p from binding WNT to active WNT/β-catenin signaling in NSCLC [92]. LINC00673 functions as a modular scaffold to strengthen the interaction between DDX3 and CK1ε, induces phosphorylation of Dvl and, therefore, promote the nuclear accumulation of β-catenin and the activation of WNT/β-catenin signaling pathway in LUAD [93]. Furthermore, the expressions of lncRNA-NEAT1, FOXO2-AS1 were positively associated with WNT/β-catenin signaling in NSCLC tissues. Knockdown of NEAT1 or FOXO2-AS1 inhibited WNT/β-catenin signaling pathway activity [91, 94, 95].

IL-6-STAT3 axis is also activated upon EGFR TKI treatment and can promote neuroendocrine differentiation in NSCLC [96]. LncRNAs such as MALAT1 and H19 are reported to, respectively, sponge miR-124, miR-17 and miR-29b-3p, subsequently activated STAT3 and promote therapy resistance [97–99]. In addition, Wang et al. showed lncRNA TNK2-AS1 was significantly upregulated in NSCLC and associated with poor survival. Mechanistically, TNK2-AS1 could interact with STAT3 to enhance its protein stability, on the other hand, STAT3 also triggers the transcription of TNK2-AS1. Thus, the positive feedback loop between TNK2-AS1 and STAT3 augmented STAT3 signaling pathway in NSCLC [100].

### The tumor microenvironment

The tumor microenvironment (TME) is a multicellular system with dynamic tumor-stromal component interactions [101, 102]. Altogether, the various stromal components such as fibroblasts, endothelial cells and infiltrating immune cells influence the response to TKI therapy.

It is well established that cancer-associated fibroblasts (CAFs) can induce EMT and TKI resistance in NSCLC cells in vitro [103, 104]. The secretion of soluble factors such as hepatocyte growth factor (HGF) promoted MET or ERK activation and subsequent EGFR TKI resistance in NSCLC cells [105, 106]. CAFs can also derive the AXL ligand growth arrest-specific protein 6 (GAS6) and enhance the expression of anti-apoptotic gene BCL2, leading to TKI resistance [26, 107].

The extracellular matrix (ECM) also interacts with NSCLC cells to promote drug tolerance. A study in 3D lung cancer cell models revealed that ECM-induced ERK and PI3K/AKT signaling lead to an EGFR TKI tolerant dormant state [108]. Low levels of SerpinB2 (a serine protease inhibitor that increase ECM stiffness), is negatively associated with gefitinib resistance in vitro. And treatment with a SerpinB2-inducing agent reversed the drug-tolerant state [108]. Moreover, Elevated levels of N-cadherin and integrinβ could promote tumor cells adhesion to ECM, thereby, promoting EGFR TKI resistance [109].

Tumor-associated macrophages (TAMs) are important infiltrating immune cells with crucial role in the development of TKI resistance [110]. Patients with increased TAM infiltration within the TME had poor outcomes [111]. Computational modelling of RNA expression in a mouse model of NSCLC revealed TAMs-secreted factors can activate various signaling pathways related to EGFR TKI resistance, including the MAPK, YAP, NF-κB, PI3K, WNT and RAS pathways [112]. Furthermore, macrophage could promote EMT through the IL-6-mediated COX2/PGE_2_/β-catenin signaling pathway [113]. Notably, a few lncRNAs such as GNAS-AS1 and XIST are reported to promote macrophage M2 polarization in NSCLC and, therefore, play a part in TAM-induced drug tolerant [114, 115].

Finally, hypoxia and the density and distribution of vasculature are closely associated with EGFR TKI resistance. Hypoxia-inducible factor 1α (HIF1α) promotes TKI resistance in a TGFα-dependent manner and increases cancer stem phenotype via IGF1R activation [116, 117]. In addition, the secretion of vascular endothelial growth factor (VEGF) by endothelial cells under hypoxia promotes angiogenesis and drug resistance [118]. Additionally, the expression of immune checkpoint programmed cell death 1 ligand 1 (PDL1) are upregulated in some EGFR-mutated or ALK-rearranged NSCLC patients. A preliminary study has found the combination of PDL1 nanobody and gefitinib displayed effectiveness of reversing the gefitinib resistance in NSCLC [119]. However, the extent of TME contributes to TKI resistance and the involvement of lncRNAs in TME-mediated resistance warrants further investigation, particularly as novel therapies that target immune and stromal cells continue to emerge. The emerging challenge is to clarify whether there is potential for therapeutic synergy between immunotherapy and targeted therapy in preventing lineage plasticity and TKI resistance.

## Targeting strategies for lineage plasticity

### Targeting epigenetic regulators

Since certain epigenetic alterations are correlated with slow-cycling drug-tolerant cells, targeting the potential epigenetic regulators serves as an important way to ameliorate lineage plasticity. Notably, preventing phenotypic switching by targeting HDAC or histone demethylases KDM5A/B and KDM6A/B have promising results in early-phase studies [120]. In NSCLC, trimethylation of lysine 9 on histone (H3K9me3) represses long-interspersed repeat elements 1 (LINE-1), thus inhibiting the expression of interferon and antiviral-activated genes to promote EGFR TKI-tolerant cell survival [121, 122]. HDAC inhibitor trichostatin A or entinostat counterbalanced the drug-tolerant cells via suppressing LINE [121]. Early-phase studies of detecting HDAC inhibitors in combination with EGFR TKI are ongoing. Inhibiting demethylases KDM6 with GSK-J4 suppressed residual persister cells in glioblastoma [123, 124]. (Table 2).

**Table 2:** Selected clinical NSCLC trials of targets and compound targeting lineage plasticity

Drug regimen	Phase	Identification	Results	Chilicaltrials.gov identifier	Refs
HDAC inhibitor					
Vorinostat + gefitinib	I/II	Relapsed/refractory advanced NSCLC	No improvement in PFS	NCT01027676	[120]
Vorinostat + erlotinib	I/II	Relapsed EGFR-mutant NSCLC	No improvement in erlotinib-resistant population	NCT00503971	[120]
Belinostat + Erlotinib	I	Molecularly unselected	Results pending	NCT01027676	[120]
SNDX-275 + Erlotinib	II	Progression on erlotinib	Results pending	NCT00750698	[120]
KDM5 and KDM6					
YUKA1, CPI-455 (KDM5A specific), KDOAM-25 (KDM5A-D specific) + gefitinib	Preclinical	EGFR-mutant NSCLC	YUKA1 in combination with gefitinib prevents drug tolerance in EFGR-mutant NSCLC		[123, 124]
CDK7/12					
SY-1365	I	Advanced solid tumors	Ongoing	NCT03134638	
AXL inhibitor					
BGB324 + erlotinib	II	Molecularly unselected NSCLC	First efficacy end point met	NCT02424617	[141]
SGI-7079 + erlotinib	Preclinical	Molecularly unselected NSCLC	Enhanced sensitivity of mesenchymal-like NSCLC cells to erlotinib		[25]
TP-0903	Ia/Ib	Advanced solid tumors	Ongoing	NCT02729298	
E-cadherin					
JMF3086	Preclinical	Molecularly unselected NSCLC	Restore sensitivity to EGFR-TKI		[130]
EZH2 inhibitor					
JQEZ5	Preclinical	EGFR or BRG1-mutant NSCLC	Decreased tumor burden in GEMM and human NSCLC models		[127, 128]
GSK343 + gefitinib	Preclinical	NSCLC	Inhibited cell viability		[129]

*NSCLC* non-small-cell lung cancer, *EGFR* epidermal growth factor receptor, *HDAC* histone deacetylase, *PFS* progression-free survival, *KDM* histone demethylase, *RXR* retinoid X receptor, *EZH2* zeste homologue 2, *IL-6* interleukin-6, *AXL* receptor tyrosine kinase, *TKI* tyrosine kinase inhibitors

**Fig. 3:**
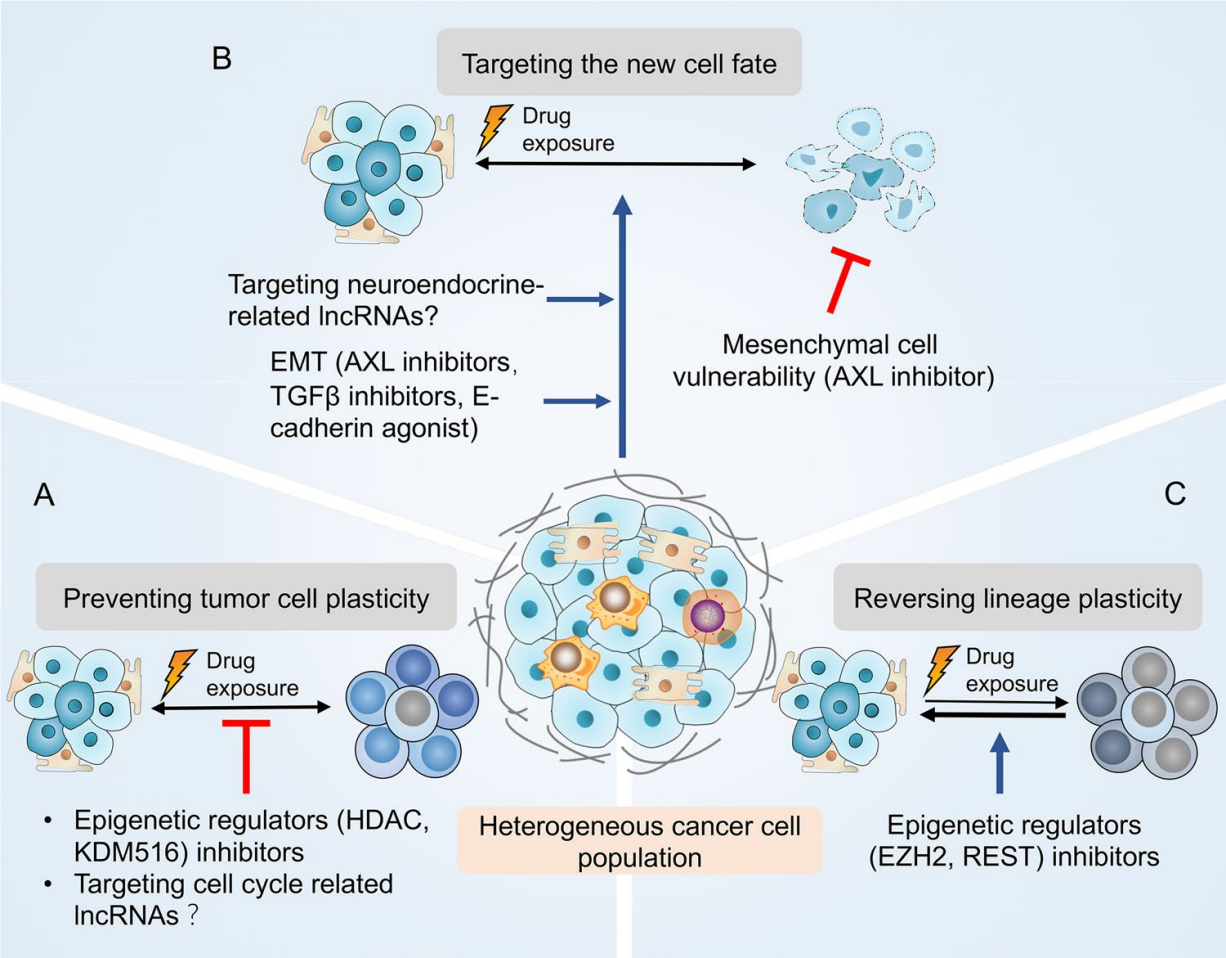
Principal strategies to target lineage plasticity in NSCLC. Three general approaches that target lineage plasticity are listed here: preventing lineage plasticity, targeting the emerging new cell identity and reversing the lineage plasticity. **a** Preventing lineage plasticity may prolong the clinical response to TKI treatment. Crucial signals and molecules that regulate the survival of slow-cycling cell, for instance, chromatin landscape remodeling modulators and cell cycle-related lncRNAs can be targeted to block tumor cellular plasticity. **b** The emerging drug-tolerant cell identity such as SCLC and epithelial–mesenchymal transition (EMT) feature can be eliminated through targeting neuroendocrine-related lncRNAs, AXL, TGFβ and E-cadherin. **c** Lineage plasticity can be reverted to resensitize NSCLC to TKI. Epigenetic regulators, such as enhancer of zeste homologue 2 (EZH2) and RE1-silencing transcription factors (REST), can be targeted for reversing lineage plasticity. *NSCLC* non-small-cell lung cancer, *SCLC* small-cell lung cancer, *TKI* tyrosine kinase inhibitor, *KDM* histone demethylase, *HDAC* histone deacetylase, *TGFβ* transforming growth factor-β

EZH2 is another druggable epigenetic regulator as mentioned previously [72]. Several phase I and phase II clinical trials are ongoing to inhibit EZH2 in lymphomas and multiple solid tumors [125, 126]. In a mouse model of human LUAD, using an open-source EZH2 inhibitor JQEZ5, Zhang et al. have established efficient antitumor effects [127, 128]. Another study has found that EZH2 inhibitor GSK343 synergy with gefitinib in NSCLC cells [129]. However, EZH2 inhibitor GSK2816126 failed in another early phase II trial in patients with similar indications [14]. Assessment of other epigenetic regulators, such as REST, are also ongoing. In addition, the dual HDAC and 3-hydroxy-3-methylgutaryl coenzyme A reductase inhibitor, JMF3086, which regulate the post-translational regulation of E-cadherin, can restore the sensitivity to first and third-generation EGFR-TKI in NSCLC [130]. (Fig. 3).

Numerous mechanistic studies support the hypothesis that targeting epigenetic regulators can synergize with TKI agents and reverse lineage plasticity in preclinical models. Nevertheless, the efficacy of epi-drugs tested in clinical trials to date has been disappointing. Overall, the epigenetic modulators exert broadening effects on cell biology and systemic physiology. Inhibitors targeting epigenetic regulators might lead to the dysregulation of cell biology [131, 132]. Therefore, novel agents are needed to target lineage plasticity more specifically. LncRNAs are excellent candidates in this respect. Several features of lncRNAs render their potential therapeutic targets in lineage plasticity-mediated TKI resistance. First, the expression of lncRNAs show strong conservation of tissue specificity [133]. Interestingly, many lncRNAs are patient and tumor specific [134]. The exclusive expression pattern of lncRNAs in specific types of tissues or cells provides an opportunity for specific regulation by lncRNA-targeting therapeutics [133, 135]. Second, chromatin modification represents an important mechanism for lncRNA, thus targeting the interaction of lncRNAs with epigenetic factors such as EZH2 can be envisioned. Third, many nuclear lncRNAs regulate neighboring gene expression in cis, thus locus-specific regulation can be achieved through lncRNA manipulation. However, the development of lncRNA therapeutics is the still in its infancy. Traditional RNAi has proven generally ineffective for lncRNA, due to their unique localization and expression [136]. Currently, antisense oligonucleotides (ASOs) and CRISPR-Cas9 are considered as promising approaches to target lncRNAs [137, 138]. Furthermore, we anticipate that future pooled CRISPR screening will be implemented to identify lineage plasticity-related lncRNAs. Nevertheless, we are beginning to understand the roles of lncRNAs in lineage plasticity. Targeting lineage plasticity associated lncRNAs in combination with TKI treatment has not been reported yet. Further translation research and clinical trials are needed.

### New cell fate management

In addition to targeting epigenetic regulators involving in lineage plasticity, it is also appealing to target the emerging drug-tolerant cell identity. There are ongoing efforts to evaluate whether NSCLC that have undergone neuroendocrine differentiation share similar treatment schedules with de novo SCLC. Notably, transformed SCLC presented sensitivity to palatium-etoposide, which makes them more similar to de novo SCLC [139]. However, it is important to note that a contrasting result came from another retrospective study, in which transformed SCLC patients still displayed higher responsive to taxanes than de novo SCLC but failed response to checkpoint inhibitor therapy [19]. Therefore, treatment regimens on transformed SCLC need to be cautiously evaluated and await further functional investigation. Interestingly, our previous study demonstrated certain non-coding RNA could facilitate the therapeutic effects of EGFR-TKI in NSCLC [140]. Our unpublished data also showed that a few lncRNAs are crucial for the transformation from NSCLC to SCLC in response to TKI treatment and could be targeted to prevent lineage plasticity. Therefore, lncRNAs involving in the histological transformation will be worthwhile areas for further investigation.

EMT represents an important process determining new cell fate of cancer. The correlation between the expression of EMT signature and receptor tyrosine kinase AXL in NSCLC indicates that AXL may represent a novel target [25]. Notably, combination of AXL inhibitor SGI-7079 with EGFR TKI erlotinib increased the sensitivity of mesenchymal-like tumor cells to erlotinib in a mouse xenograft NSCLC model [25]. Preliminary findings also suggest that BGB324, the first AXL-specific-molecule inhibitor approved for clinic, can be safely administered, resulting in disease stabilization in a group of NSCLC patients [141].

## Conclusion

Along with genetic alterations, lineage plasticity has recently considered to play a key part in the development of TKI resistance in NSCLC. Despite the functions of lncRNAs in cancer have been extensively studied in the past few years, we are beginning to understand the implications of lncRNA in TKI resistance in NSCLC, particularly in regulating lineage plasticity-mediated drug escape. A better understanding of the deregulated lncRNA involved in lineage plasticity will shed light on the complexity of the molecular alterations underlying TKI resistance. Recently, CRISPR-based screening has successfully identified many cancer-promoting lncRNAs. The simplicity, low cost and flexibility of pooled CRISPR screening brings transcriptome-wide screens within reach of the average molecular oncology laboratory. We anticipate that future pooled CRISPR screening will be implemented to identify lineage plasticity-related lncRNAs. The current strategies to target lineage plasticity mainly include targeting the epigenetic and transcriptional alterations. However, there remains many challenges ahead to better understand the process. First, the molecular determinants and biomarker for tumor cell phenotypic plasticity upon TKI treatment needs to be better understood. Second, the heterogeneity should be taken into account when targeting the residual tumor cells. Finally, further investigations such as CRISPR screening are required to discover lineage plasticity-related lncRNAs, and use lncRNAs as novel diagnosis and treatment modalities. Overcoming the obstacles will enable us to implement personalized medicine in the treatment of TKI resistance in NSCLC patients.

## Data Availability

All data generated during this study are included in this published article.

## Abbreviations

NSCLC: Non-small-cell lung cancer
lncRNA: Long non-coding RNA
TKI: Tyrosine kinase inhibitors
EGFR: Epidermal growth factor receptor
BRAF: Serine/threonine-protein kinase b-raf
ALK: Anaplastic lymphoma kinase
TME: Tumor microenvironment
EZH2: Enhancer of zeste homolog 2
ATL-1: Atractylodes macrocephula Koidz atractylenolide 1
STAT3: Signal transducer and activator of transcription
PI3K: Phosphatidylinositol 3-kinase
SCLC: Small-cell lung cancer
EMT: Epithelial-to-mesenchymal transition
NCAM1: Neural cell adhesion molecule 1
RB1: RB transcriptional corepressor 1
TP53: Tumor protein p53
CRPC: Castration-resistant prostate cancer
AR: Androgen receptor
NEPC: Neuroendocrine prostate cancer
ZEB: Zinc finger E-box-binding homeobox
TGFβ: Transforming growth factor-β
IL-6: Interleukin 6
LSD1: Lysine-specific demethylase 1
DNMT1: DNA methyltransferase 1
CDKN1A: Cyclin-dependent kinase inhibitor 1A
YAP1: Yes-associated protein 1
PRC2: Polycomb repressive complex 2
REST: RE1-slienicng transcription factor
TFBS: Transcription factor biding site
LUAD: Lung adenocarcinoma
E2F1: E2F transcription factor 1
INSM1: Insulinoma-associated-1
ASCL: Achaete-scure complex homolog 1
ASH1: Achaete-scute homolog 1
CAF: Cancer-associated fibroblast
HGF: Hepatocyte growth factor
MET: Mesenchymal–epithelial transition factor receptor
GAS6: Growth arrest specific 6
EGF: Epidermal growth factor
HB-EGF: EGF-like growth factor
ECM: Extracellular matrix
CXCL12: CXC-chemokine ligand 12
TAM: Tumor-associated macrophage
MAPK: Mitogen activated kinase-like protein
NF-κB: Nuclear factor kappa B
RAS: Resistance to audiogenic seizures (RAS)
COX2: Cytochrome coxidase subunit II
PGE_2_: Nr5a1 enhancer region in intron 6
HIF1α: Hypoxia-inducible factor 1α
VEGF: Vascular endothelial growth factor
PDL1: Programmed cell death 1 ligand 1
HDAC: Type-2 histone deacetylase 2
H3K9me3: Trimethylation of lysine 9 on histone
